# Blended vs. face-to-face cognitive behavioural treatment for major depression in specialized mental health care: study protocol of a randomized controlled cost-effectiveness trial

**DOI:** 10.1186/s12888-014-0290-z

**Published:** 2014-10-18

**Authors:** Lisa C Kooistra, Jenneke E Wiersma, Jeroen Ruwaard, Patricia van Oppen, Filip Smit, Joran Lokkerbol, Pim Cuijpers, Heleen Riper

**Affiliations:** Faculty of Psychology and Education, Department of Clinical Psychology, VU University Amsterdam, Van der Boechorststraat 1, BT 1081 Amsterdam, the Netherlands; EMGO institute for Health Care and Research, VU University Medical Centre, Van der Boechorststraat 7, BT 1081 Amsterdam, the Netherlands; Department of Psychiatry, GGZ inGeest and VU University Medical Centre, P.O. Box 7057, Amsterdam, MB 1007 the Netherlands; Trimbos Institute, P.O. Box 725, Utrecht, AS 3500 the Netherlands; Department of Epidemiology and Biostatistics, VU University Medical Centre, P.O. Box 725, Utrecht, AS 3500 the Netherlands; Leuphana University, Innovation Incubator, Division Health Trainings online, Rotenbleicher Weg 67, Lüneburg, 21335 Germany

**Keywords:** Depression, Internet-based treatment, Cognitive behavioural therapy, Blended treatment, Cost-effectiveness, Outpatients, Specialized mental health care, Pilot, Randomized controlled trial

## Abstract

**Background:**

Depression is a prevalent disorder, associated with a high disease burden and substantial societal, economic and personal costs. Cognitive behavioural treatment has been shown to provide adequate treatment for depression. By offering this treatment in a blended format, in which online and face-to-face treatment are combined, it might be possible to reduce the number of costly face-to-face sessions required to deliver the treatment protocol. This could improve the cost-effectiveness of treatment, while maintaining clinical effects. This protocol describes the design of a pilot study for the evaluation of the feasibility, acceptability and cost-effectiveness of blended cognitive behavioural therapy for patients with major depressive disorder in specialized outpatient mental health care.

**Methods/design:**

In a randomized controlled trial design, adult patients with major depressive disorder are allocated to either blended cognitive behavioural treatment or traditional face-to-face cognitive behavioural treatment (treatment as usual). We aim to recruit one hundred and fifty patients. Blended treatment will consist of ten face-to-face and nine online sessions provided alternately on a weekly basis. Traditional cognitive behavioural treatment will consist of twenty weekly sessions. Costs and effects are measured at baseline and after 10, 20 and 30 weeks. Evaluations are directed at cost-effectiveness (with depression severity and diagnostic status as outcomes), and cost-utility (with costs per quality adjusted life year, QALY, as outcome). Costs will encompass health care uptake costs and productivity losses due to absence from work and lower levels of efficiency while at work. Other measures of interest are mastery, working alliance, treatment preference at baseline, depressive cognitions, treatment satisfaction and system usability.

**Discussion:**

The results of this pilot study will provide an initial insight into the feasibility and acceptability of blended cognitive behavioural treatment in terms of clinical and economic outcomes (proof of concept) in routine specialized mental health care settings, and an indication as to whether a well-powered clinical trial of blended cognitive behavioural treatment for depression in routine practice would be advisable. This will be determined based on the perspective of various stakeholders including patients, mental health service providers and health insurers. Strengths and limitations of the study are discussed.

**Trial registration:**

Netherlands Trial Register NTR4650. Registered 18 June 2014.

## Background

Depression is a highly prevalent disorder [1,2]. The World Health Organization (WHO) has predicted that by 2030 depressive disorders will have the highest disease burden in developed countries [3]. As a result, depression is associated with considerable costs [4-6]. These costs stem from various sources, such as direct medical costs of health care uptake, as well as non-medical costs associated with patients' out-of-pocket expenses in the context of receiving treatment. In addition, production losses related to reduced efficiency at work and absenteeism constitute indirect non-medical costs [4].

In addition, health care budgets are shrinking, and mental health care resources, such as the availability of qualified therapists, are limited [7].

It is therefore of major importance to foster the development and implementation of depression treatments that are both evidence-based and cost-effective [4,8]. In addition, a much more efficient health care system is needed to ensure that appropriate treatment can still be delivered to those who need it.

Online treatment for depression has the potential to provide both a clinically effective [9] and an efficient approach to the reduction of treatment costs [10]. Several meta-analyses have shown that online treatment for depression is effective when compared with non-intervention, both in the short and long term, especially for cognitive behavioural therapy (CBT) [9,11-13]. In addition, online treatment supported by a professional and face-to-face treatment have been shown to be equally clinically effective [9,14]. However, the cost-effectiveness of online treatment is currently less well-documented. Preliminary evidence shows that when society is willing to invest a modest level of resources in alleviating depressive symptoms, treatment will be cost-effective when compared with no intervention [15] and that online treatment can be cost-effective when compared with face-to-face treatment [16-19].

Despite promising results, implementation and use of online treatment in routine practice has been slow to get off the ground. In the Netherlands, for example, it was recently estimated that only 1 to 5% of patients in mental health care are being treated online [7]. This was primarily attributed to the fact that stakeholders, such as patients and therapists, seem sceptical about online treatment and the potential costs and benefits are not yet clear to them [7]. Blended treatment could bridge the gap between stand-alone online treatment and traditional face-to-face therapy. This type of treatment involves a combination (blending) of face-to-face treatment with Internet sessions into one integrated treatment in such a way that it can be delivered in routine care settings [20]. It can build on established face-to-face treatment protocols, rather than making a full transition to a new online infrastructure. Therefore, if proven effective, it could garner greater acceptance among various stakeholders such as health services, therapists, and patients compared with stand-alone online treatment.

In terms of cost-effectiveness, blended treatment could potentially reduce the direct medical costs of treatment per patient compared with treatment as usual, by replacing a portion of the face-to-face sessions with more efficient online sessions. By reducing the number of one-on-one sessions, blended treatment also provides an opportunity for therapists to take on more patients. This could in turn improve access to treatment for those suffering from depression. Furthermore, it could contribute to lowering direct non-medical costs of treatment, such as travel time for patients, as they can access information and exercises any time at home via an online platform [21].

Another possible strength of blended treatment could be that online treatment sessions ensure structured delivery and monitoring of the core treatment information and exercises, as a complement to face-to-face sessions.

In the face-to-face sessions, therapists can in turn offer customized treatment by responding to the patient's needs, problems or wishes in real time [22]. Furthermore, encouraging patients to take an active role in treatment might lead to improved self-management skills [23,24].

However, having a functional (tablet) computer with an Internet connection and learning to work with the required hardware, software and online environment requires both a financial and time investment from all parties involved. This might prove to be burdensome and more costly than warranted by the savings made.

The number of studies that have investigated clinical and cost-effectiveness of blended treatment for adult depression is still limited. Preliminary results suggest that blended treatment can be effective in diminishing depressive symptoms [22,25-28]. Most studies focused on evaluating clinical outcomes where online CBT is provided in addition to care-as-usual, either by a GP in primary care [25-27] or by mental health care providers in primary and secondary care settings [28]. However, in terms of cost-effectiveness, it is debatable whether this blended approach is advisable, since treatment intensity is increased without reducing the number of face-to-face sessions. The uncontrolled study by Månsson et al. [22] provides support for the proposition that face-to-face and online sessions can be combined into a single eight to nine-week blended CBT protocol. When offered to people with moderate anxiety or depression in the general population (n = 15), large within-group effect sizes were found at both short and long-term follow-up.

The present trial will focus on blended CBT for adults with major depressive disorder in outpatient specialized mental health care settings. This specific target group was chosen because in the Netherlands, this sector accounts for more than half of health care costs related to depression [29]. Therefore, improving efficiency and cost-effectiveness is particularly relevant in this sector. With the criterion of cost-effectiveness in mind, an integrated approach to blended CBT was chosen, rather than an add-on approach. Face-to-face and online sessions are combined in such a way that blended CBT involves less therapist time than regular face-to-face CBT. The blended CBT protocol was developed by our project group as part of a preliminary feasibility study.

### Aims

In the present paper, we present the study protocol for a pilot cost-effectiveness randomized controlled trial. The primary goal of the study is to assess the probability that blended cognitive behavioural treatment (bCBT) is more cost-effective compared with regular face-to-face cognitive behavioural therapy (CBTAU). Because the content of treatment remains unchanged, blended cognitive behavioural treatment is expected to maintain the clinical effectiveness associated with face-to-face CBT [30]. This expectation is in line with findings indicating that face-to-face CBT and stand-alone online CBT supported by a professional appear to be equally clinically effective [9,14].

The results of this pilot study will provide 1) an initial insight into the feasibility and acceptability of ‘blended treatment’ in terms of clinical and economic outcomes (proof of concept), 2) an indication as to whether blended treatment will add value when implemented in routine specialized mental health care settings, and 3) a good indication as to whether an adequately powered clinical and economic randomized controlled trial of blended treatment for depression in routine practice is advisable and feasible from the perspective of different stakeholders including patients, mental health service providers and health insurers.

## Methods

### Study design

The pilot study is designed as a randomized controlled trial with two parallel groups (N = 150). Participants will be randomized to either blended CBT (bCBT) or usual face-to-face CBT (CBTAU). Participants in both groups will complete assessments at baseline and at three fixed 10-week intervals after the first treatment session (Week 10, 20 and 30).

**Figure 1:**
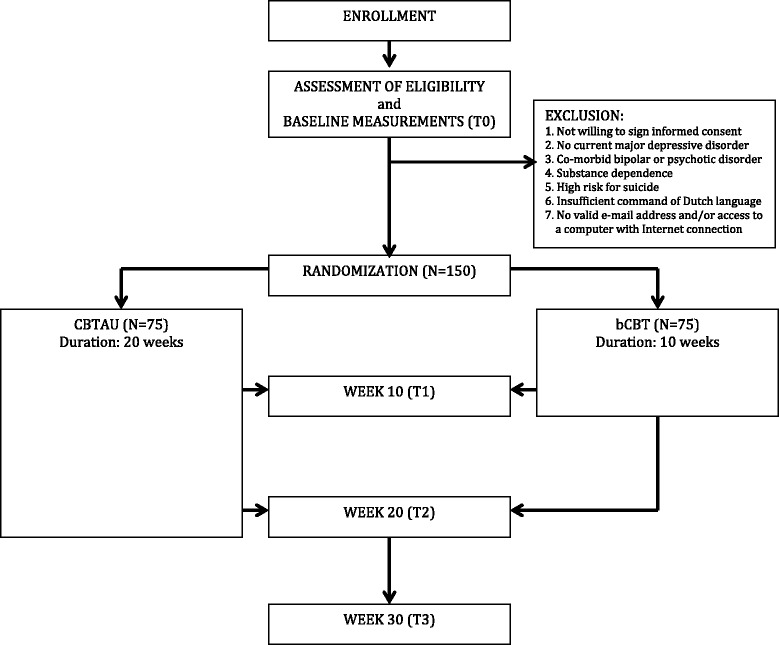
**Flowchart of the study design.**

The design incorporates the ISPOR RCT-CEA Task Force recommendations for cost-effectiveness analyses alongside clinical trials and the ISPOR Consolidated Health Economic Evaluation Reporting Standards (CHEERS) [31-33]. The protocol for this study has been approved by the Medical Ethics Committee of the VU University Medical Centre (Registration number 2014.191). Written informed consent will be obtained from all participants. Figure 1 displays the flowchart of the study design.

### Participants

#### Inclusion criteria

Patients aged 18 and older are eligible to participate if they meet the criteria for a DSM-IV diagnosis of Major Depressive Disorder (MDD). The MINI international Neuropsychiatric Interview plus (M.I.N.I. plus), a structured diagnostic interview performed by a trained researcher, will be used to assess these inclusion criteria [34,35].

#### Exclusion criteria

Patients are excluded from the study if they a) do not have adequate proficiency in the Dutch language, both verbal and written, b) do not have a valid e-mail address and a (tablet) computer with Internet access, c) suffer from one or more of the following disorders: a psychotic disorder, bipolar disorder and/or substance dependence, d) are identified to be at high risk for suicide. The MINI plus [34,35] will be used to assess whether the exclusion criteria c and d apply. High risk of suicide is assessed through Questions 3 to 6 of Section C of the MINI plus. Comorbid disorders other than psychotic and bipolar disorders are allowed, as is psychopharmacological treatment.

Excluded participants will be directed to treatment options within the participating specialized mental health care centre. For respondents with a heightened suicide risk, the principal investigator will inform the professional responsible for treatment immediately via telephone and e-mail.

### Recruitment

Patients will be recruited upon registration at the mood disorder departments of two specialized mental health care centres in the Netherlands. In accordance with the standard procedure within the centres, all newly registered patients first undergo an intake interview by an experienced clinician (psychiatrist or clinical psychologist), after which diagnosis and treatment is established and discussed with the patient. During this conversation, patients are informed about the present study.

After intake, the research coordinator will phone eligible patients who are willing to participate in the study and inform them about the trial. Interested patients will then receive an information brochure and an informed consent form via e-mail and will be invited to take the baseline M.I.N.I. plus interview at the health care centre. During this interview, a trained researcher will confirm the primary diagnosis of Major Depressive Disorder and assess comorbidity. If patients are willing and eligible to participate, written informed consent will be requested.

### Randomization and blinding

Once the signed informed consent form has been received and all baseline assessments have been completed, patients will be randomly assigned to either bCBT or CBTAU. Randomization will be performed at individual level, based on an allocation ratio of 1:1. The randomization will be stratified by site by an independent researcher using a computer generated random number table [36]. Group allocation cannot be blinded to patients and therapists. However, the assessors conducting the diagnostic interviews will be blinded to allocation, in accordance with the CONSORT guidelines [37,38].

### Interventions

Cognitive behavioural therapy (CBT) will be provided in both treatment conditions. The CBT protocol consists of psycho-education, behavioural activation, cognitive therapy and relapse prevention [39]. In addition, depressive symptoms will be monitored throughout the treatment. CBT is one of the recommended treatments for depression in the Netherlands, set out in the multidisciplinary guidelines for depression [29]. CBT has been extensively studied over recent decades and favourable effects with regard to clinical outcomes have been found consistently [30].

All participating therapists are trained in CBT and have a minimum of two years work experience in Dutch mental health care. Therapists work with both treatment groups. Prior to the study, they will be trained in the blended CBT protocol. During the trial, therapists will attend peer group supervision meetings every other week. The supervision meetings are guided by the head researcher at the centre (an experienced psychologist) and the research coordinator.

Pharmacotherapy will be offered when necessary by a psychiatrist. The same medication regimes will be administered for both conditions throughout the study.

#### Cognitive behavioural treatment-as-usual (CBTAU)

In the CBTAU condition, patients receive, on average, twenty 45-minute sessions of face-to-face CBT over 20 weeks (one session per week). Therapists follow an established face-to-face CBT protocol [39] that contains the components referred to above. Therapists are required to include all four elements of CBT, but are free to decide how many sessions are spent on each module.

All individual sessions should involve monitoring of depressive symptoms, addressing the week gone by and current issues, and discussing information and homework exercises for the previous week and the week to come. Sessions will be concluded with a summary and evaluation. Patients receive their homework exercises on paper.

#### Blended cognitive behavioural treatment (bCBT)

For the bCBT group, the existing CBT protocol [39] is divided over ten 45-minute face-to-face sessions and nine online sessions. The treatment will be delivered over a period of ten weeks (one face-to-face and one online session per week). Treatment starts and ends with a face-to-face session. The therapist follows a fixed treatment protocol. The blended face-to-face sessions are structured in a similar way to the regular face-to-face sessions, leaving the therapist room to respond to the individual patient’s needs in each session. During the sessions, the patient and therapist are logged in on the personal patient environment in the online platform.

The online platform is a secure web-based environment (Minddistrict; www.minddistrict.com). Patients and therapists access this platform with a personalized login. The website offers information that reinforces and develops on the content of the face-to-face sessions. A short video fragment is included in each online session in which a therapist explains the theory in lay terms.

In addition, patients use the website to complete homework exercises, such as monitoring their activities, feelings, thoughts and behaviour. Testimonials from two fictional patients are provided to give insight in how the exercises can be executed.

Therapists monitor their patients’ online progress and provide feedback each week before the next face-to-face session. The feedback messages take approximately 15 minutes to write and are sent on the online platform to ensure secure communication.

On completion of treatment, patients can continue to access the online treatment platform to reread information and look up homework exercises, such as the relapse prevention plan.

### Assessments

The health-economic analyses combine clinical outcomes (i.e., depression severity and diagnosis of major depressive disorder) and quality adjusted life years (QALYs) with cost estimates (both direct and indirect costs).

Data are collected at four intervals: at baseline (T0), 10 weeks after commencing treatment (T1), 20 weeks after commencing treatment (T2) and 30 weeks after commencing treatment (T3). All questionnaires are administered online. Diagnostic interviews are administered either face-to-face at the treatment location or via telephone, based on the patient’s preference. Table 1 provides an overview of the measures that are used at each time point.

**Table 1:** **Overview of measures administered at each assessment interval**

**Questionnaire**	**Aim**	**Baseline (T0)**	**Week 10 (T1)**	**Week 20 (T2)**	**Week 30 (T3)**
**Primary outcomes**					
IDS-SR	Depression severity	x	x	x	x
MINI plus full	Diagnostic interview	x			x
MINI plus; Section A			x	x	
EQ-5D-3L	Health-related QoL	x	x	x	x
SF-36	Functional impairment	x	x	x	x
TiC-P	Health care utilization	x	x	x	x
**Other variables of interest**					
General patient characteristics		x			
A priori treatment preference		x			
Mastery Scale	Locus of control	x	x	x	x
WAI-SR	Therapeutic alliance		x		
CCL-D	Depressive cognitions	x	x	x	
CSQ	Treatment satisfaction				x
SUS (bCBT only)	System usability			x	

bCBT: Blended Cognitive Behavioural Therapy; CCL-D: Cognition Checklist-Depression scale; CSQ: Client Satisfaction Questionnaire; EQ-5D-3L: EuroQol; IDS-SR: Inventory of Depressive Symptomatology, Self-Report version; MINI plus: Mini International Neuropsychiatric Interview Plus; QoL: Quality of Life; SF-36: 36-item Short Form Health Survey; SUS: System Usability Scale; TiC-P: Trimbos and iMTA questionnaire on Costs associated with Psychiatric illness; WAI-SR: Work Alliance Inventory-Short Revised.

### Clinical outcome measures

#### Diagnosis of depression

The Mini-International Neuropsychiatric Interview (MINI) [34] is a brief clinician-administered structured diagnostic interview for assessing the presence of psychiatric disorders as per *the Diagnostic and Statistical Manual of Mental Disorders* (*Fourth edition; DSM-IV*) and *the International Classification of Diseases, Tenth Revision* (*ICD-10*). The interview has been validated in Dutch [35].

At T0, the MINI will be administered to assess past and present MDD (including number of episodes, age of onset, and duration of current episode) and comorbid disorders. At T1 and T2, only Section A of the MINI will be administered to assess the presence of MDD. At T3, the full MINI will be administered again to assess current MDD and comorbid disorders.

#### Severity of depressive symptoms

The 28-item self-report version of the Inventory of Depressive Symptoms (IDS-SR) will be used to measure the severity of depressive symptoms [40]. The IDS-SR will be administered at all time points (T0-T3). Each item has four response categories, with scores ranging from 0 to 3. The total score ranges from 0 to 84, with higher scores being indicative of a higher severity of depressive symptoms. The IDS-SR has highly acceptable psychometric properties and has been proven to be sensitive to treatment effects in depressed outpatients [41-43]. Patients with a ≥50% symptom reduction on the IDS-SR will be deemed to be treatment responders. Remission is defined as an IDS-SR score of 13 or less, which is indicative of no depression severity [42].

A shortened version of the IDS-SR, the 16-item Quick Inventory of Depressive Symptomatology (QIDS-SR) [44] is administered within both treatment conditions to monitor the depressive symptom change trajectory more closely. Patients are encouraged to complete the QIDS-SR on a weekly basis. The questionnaire has highly acceptable psychometric properties [44].

### Measures for quality adjusted life years

#### Quality of life

The EQ-5D-3L [45,46] will be administered at all time points (T0-T3) to assess quality of life. This questionnaire consists of five items and a visual analogue scale (VAS). Each item has three response categories, ranging from 1 (no problems) to 3 (severe problems). The items provide insight on perceived problems in the areas of mobility, self-care, usual activities, pain/discomfort and anxiety/depression. On the VAS, patients can rate their health state from 0 (worst possible health state) to 100 (best possible health state).

Based on the combination of answers on the five items, the questionnaire differentiates between 243 distinct health states (for example 11231), for which pre-determined values (utility scores) have been set, anchored at 0 (death) and 1 (good health) [46]. The utility score is then used to compute quality-adjusted life years (QALYs). This is done by weighing the amount of time spent in a particular health state against its corresponding utility score [47,48].

#### Functional impairment

The 36-item Short Form Health Survey (SF-36) is used to measure functional impairment and to assess health-related quality of life [49-52] using the Brazier algorithm, which maps utilities on 249 health states [53] which can be extracted from six SF-36 items, namely physical functioning, role limitation due to physical problems, bodily pain, general health, social functioning, role limitations due to emotional problems, mental health and vitality. Thus, the SF-36 offers a second way to measure QALY changes [48].

### Cost calculations

The economic evaluation will be conducted from both the health care and the societal perspective. Therefore, both medical costs and non-medical costs (direct and indirect) are calculated.

#### Medical costs

The medical costs that arising from health care uptake are assessed using Part 1 of the Trimbos/iMTA questionnaire for Costs Associated with Psychiatric Illness (TiC-P) [54]. The questionnaire will be administered at all time points (T0-T3). The TiC-P is the most widely used health care service recipient interview used for economic evaluations in the Netherlands. Part 1 consists of 23 questions on health care uptake among relevant health care providers in the past four weeks, such as medication taken and the number of contacts within the mental health care settings. Patients indicate whether they visited a health care provider and, if so, how often. To determine the costs associated with such visits, care consumption is multiplied by the Dutch standard cost price (i.e. the full economic cost prices) as outlined in the guideline for economic evaluation [55]. All costs will be indexed for the reference year 2014.

#### Intervention costs

Apart from health care uptake, the costs of developing and maintaining the online bCBT platform will be taken into account, as well as the costs of weekly therapist feedback in the bCBT condition.

#### Non-medical costs

Direct non-medical costs, or *patients’ out-of-pocket expenses*, such as the cost of traveling to attend health services and the costs associated with the time spent by patients travelling, waiting and receiving treatment, are determined in accordance with the standard cost prices as listed in the relevant Dutch guidelines for economic evaluation [55].

Indirect non-medical costs, stemming from productivity losses due to absenteeism and lower efficiency levels while at work, are assessed with the second part of the Trimbos/iMTA questionnaire for Costs associated with Psychiatric Illness (TiC-P) [54], which evaluates productivity losses in the past four weeks. It will be administered at all time points (T0-T3). The questionnaire consists of 12 questions that focus on the number of days absent from work and the number of days with reduced efficiency due to feeling ill. The costs of productivity losses will be based on the gender and age specific friction costs, as outlined in the Dutch guideline for costing [55].

### Other variables of interest

#### General patient characteristics

Information on general demographic variables such as age, gender, marital status, income and educational level and data on patients’ professional and personal computer use will be collected at baseline.

#### Treatment preference

In order to determine whether this influences treatment outcomes, patients are asked what their treatment preference would have been, before they are informed about their actual treatment allocation. The possible answers are preference for bCBT or a preference for CBTAU. Whether or not the preference matches the treatment allocation will be taken into account.

#### Mastery

The 5-item version of the Mastery Scale [56] is administered at all time points (T0-T3) to assess changes in locus of control. Item scores range from 1 to 5, resulting in total scores ranging from 5 to 25, with higher scores being indicative of a higher degree of perceived control. The scale has good psychometric properties [56].

#### Work alliance

The Revised Short Version of the Work Alliance Inventory (WAI-SR) [57,58] is administered to both patients and therapists in order to determine the quality of the therapeutic alliance at T1. Item scores range from 1 to 5. The total score ranges from 12 to 60, with higher scores reflecting a better alliance between therapist and patient. The questionnaire has highly acceptable psychometric properties [58].

#### Depressive cognitions

The 14-item depression scale of the Cognition Checklist [59] will be administered at all time points (T0-T3) to assess the frequency of automatic thoughts relevant to depression. The items assess the frequency with which a thought typically occurs. Item scores range from 0 to 4. Total scores range from 0 to 56 with higher scores being indicative of more frequent negative depressive cognitions. The convergent and discriminant validity of the subscale has been found to be high [60].

#### Treatment evaluation

The 8-item Client Satisfaction Questionnaire-8 (CSQ-8) [61] will be administered at T3 to determine general satisfaction with treatment. High internal consistency has been reported [62]. In addition, the 10-item System Usability Scale (SUS) [63] will be administered to participants randomized to bCBT at T2 in order to evaluate the online blended treatment platform. The questionnaire has been found to be reliable [64].

#### Process data

Data for process analyses will be obtained from the administration offices of participating mental health care institutions and through usage statistics on the online platform. In addition, we will perform qualitative interviews with a random selection of ten patients per treatment group after Week 30. The interviews will focus on the feasibility and usability of the CBT treatment provided.

In the process analyses, we will also include the aspects that serve as input for the study flow chart in accordance with the CONSORT guidelines [36,38], such as exclusions, as well as study and treatment withdrawal rates for each group, including reasons. In addition, we will assess:

- The extent to which other treatments were provided in parallel with bCBT or CBTAU, such as pharmacotherapy, and the nature of this treatment.
- The total number of online sessions and/or face-to-face contacts and cancellations (amount of therapy received);
- Time investment by both the patient and the therapist. In the bCBT group, time investment can be calculated based on the number of face-to-face contacts and the amount of time spent working on the online platform. In the CBTAU group, time investment will be estimated based on the number of face-to-face contacts.
- Adverse effects.

### Sample size

Given the nature of our pilot study, we chose to conduct a cost-effectiveness study, in which we focus on the probability of cost-effectiveness of bCBT in comparison with CBTAU for various ‘willingness to pay’ ceilings, i.e., the cost-effectiveness acceptability curve (CEAC; Fenwick et al. 2001). The probabilistic assessment does not entail hypothesis testing. Instead, the outcomes will be presented as the likelihood (in %) that bCBT is superior to CBTAU in terms of cost-effectiveness [18].

Through a simulation study, we determined the impact of various sample sizes on the stability of the CEAC. Using realistic fixed estimates of the mean and standard deviation of effects and costs at population level (based on Hakkaart-van Roijen et al., 2006), we simulated a large number of trials in which we systematically varied sample sizes between n = 10 to n = 500 per group (n = 10, 25, 50, 75, 150, 500). As expected, the stability of the CEAC improved with increasing sample size. Below n ≤50, probability estimates were highly unstable. At a sample size of n = 75 per group, however, we found probability estimates to converge to acceptable 75% percentile ranges within the relevant range of willingness to pay ceilings. Therefore, for this study, we settled for a sample size of N = 150 (n = 75 per group).

### Statistical analyses

#### Primary analyses

A cost-effectiveness analysis (CEA) and cost-utility analysis (CUA) will be conducted from both the health care perspective and the societal perspective. Therefore, we will take direct medical costs, direct non-medical costs and indirect medical costs into account. In addition, a budget impact analysis (BIA) will be based on a health-economic modelling study based on Mauskopf’s recommendations [32]. Based on the BIA, expected costs to the public purse, the health care insurer and the service-provider will be estimated.

#### Cost-effectiveness and cost-utility analyses

The *cost-effectiveness analysis* (CEA) will be based on treatment response. Analyses will be conducted for both treatment response, defined as a 50% pre-post reduction of IDS-SR depressive symptoms [40,42], assessed as the DSM-IV depression status at 30 weeks, as measured by the MINI plus interview [35].

The *cost-utility analysis* (CUA) will be conducted using quality adjusted life years (QALYs) as a generic measurement of health gains, based on EQ-5D [45,46] and SF-36 data [49,50,53].

In order to obtain the costs per treatment responder and the costs per QALY gained, the incremental cost-effectiveness ratio (ICER) will be computed using the bootstrap method. The simulated ICERs will then be projected onto the cost-effectiveness plane and presented as acceptability curves.

For decision-making purposes, the ICER acceptability curve will be plotted using various willingness-to-pay (WTP) ceilings, which supports analysis as to whether the blended intervention offers good value for money, compared with CBT treatment as usual. One-way sensitivity analyses directed at uncertainty in the main cost drivers will be performed to assess the robustness of our findings across a range of likely values for those parameters.

#### Budget impact analysis

To assess the impact of blended CBT for depression compared with CBT as usual on health care budgets, a *budget impact analysis* (BIA) will be conducted as outlined by Mauskopf et al. [32]. The BIA will assess the impact on 1) wider society (including productivity losses), 2) the public purse, and 3) health insurers and health care service providers. When taking the public purse and health insurance companies into account, the focus will be restricted to direct medical costs. For each angle, we assess costs when 10%, 20%, 30% and 100% of the target group receive bCBT. These scenarios will be compared with the baseline scenario, reflecting current care, where 0% of the target group is offered bCBT.

Regarding the costs to wider society, we will assess the costs of offering the health care interventions, as well as the interventions offered in routine specialized mental health care for this particular target group, patients’ out of pocket expenses, and costs arising from productivity losses.

The BIA will be conducted using a health economic (Markov cohort) simulation model, called DepMod [16]. The model is based on the epidemiology of depressive disorder as obtained from the population-based NEMESIS psychiatric cohort study [65]. DepMod compares two health care systems: care-as-usual for depressive disorder and the alternative health care scenario with blended cognitive behavioural treatment. Costs and effects will be modelled for the short term (1 year) and longer term (5 years), at national level, as well as those incurred by the health care provider and health insurance company. Long-term costs and effects will be discounted in accordance with the Dutch guidelines [55]. DepMod involves extensive sensitivity analyses that cover cost, effect and discounting parameters simultaneously [66].

### Explorative analyses

The analyses will be conducted in accordance with the intention to treat (ITT) principle. Missing data will be imputed using state of the art imputation methods, a reliable method for handling missing values [35]. In addition, per protocol analyses will be conducted; if equal clinical effects are found, such analyses are more conservative than ITT [67].

Outcomes for continuous variables, such as severity of depressive symptoms and mastery, at T1, T2 and T3 (Week 10, 20, and 30) are estimated for descriptive purposes through mixed-model analyses (MM), with participants as random effects, and time (T1-T3), group (bCBT vs. CBTAU) and time x group as fixed effects, with baseline scores as a single covariate. To assess the magnitude of treatment effects, within and between-group Cohen’s *d* effect sizes [68] for each time point are calculated by dividing MM parameter estimates of fixed effects at each post-treatment assessment by the pooled standard deviation of outcome measurements at baseline (T0). Effect sizes under 0.2 are deemed to be small, 0.5 are deemed moderate and 0.8 are deemed to be large [68].

## Discussion

Major Depressive disorder is a highly prevalent disorder, with a major impact on personal, professional and family life and is associated with considerable medical and non-medical costs. In order to ensure the availability of affordable evidence-based treatments in the future, investing in the development of cost-effective treatments is warranted. This is especially relevant in specialized mental health care, as treatment in this sector tends to be intensive and of a long duration.

Blended care may offer a way to improve the cost-effectiveness of depression treatment. This type of treatment combines elements of online and face-to-face treatment, with the goal of diminishing the number of face-to-face sessions needed to deliver the treatment protocol.

### Strengths and weaknesses

In the proposed study, integrated blended cognitive behavioural treatment will be compared with face-to-face cognitive behavioural treatment for major depression. To the best of our knowledge, this is one of the first studies to evaluate this type of blended treatment in an outpatient specialized mental health care setting. Therefore, the trial is designed as a cost-effectiveness study to assess the probability that blended CBT is cost-effective in comparison with face-to-face CBT in the short term (30 weeks) by way of proof-of-concept. The results of the study provide information on the possible strengths and benefits of blended CBT and will support decision-making on whether an adequately powered clinical and economic randomized controlled trial of blended treatment for depression in routine practice is advisable and feasible. By carrying out budget impact analyses, we aim to inform stakeholders from various perspectives, including patients, mental health service providers and health insurers.

In order to facilitate the decision-making process, the study is designed to closely adhere to established procedures in routine practice in outpatient specialized mental health care. This is reflected in the decision to deliver blended CBT over a ten-week period, while adhering to the usual time frame of 20 weeks for the face-to-face CBT. Two factors explain this choice in relation to blended CBT. First, providing online and face-to-face sessions within the same week is common practice in the context of guided online treatment. Second, a recent meta-analysis by Cuijpers et al. [69] showed that intensifying face-to-face treatment from one to two sessions a week might be beneficial in terms of clinical results, with an increase in effect size of *g* = 0.45. In the proposed study, the reason not to match the intensity of treatment as usual is that this would deviate from current practice, therefore undermining the goal of the proposed study. As a result, any clinical results will be harder to attribute to the blended intervention. However, the aim of this pilot study is to gain insight into the implementation value of blended CBT, rather than clinical effectiveness.

In summary, blended cognitive behavioural treatment (CBT) for major depression might be more cost-effective than face-to-face CBT as usual, while maintaining clinical effects. The proposed study is designed to contribute to the body of knowledge on the possible value of blended treatment for depression when applied in outpatient specialized mental health care. It aims to underpin future decisions on whether further research on blended treatment for depression in routine practice is advisable and feasible.

### Trial status

The trial is in the on-going recruitment phase.
